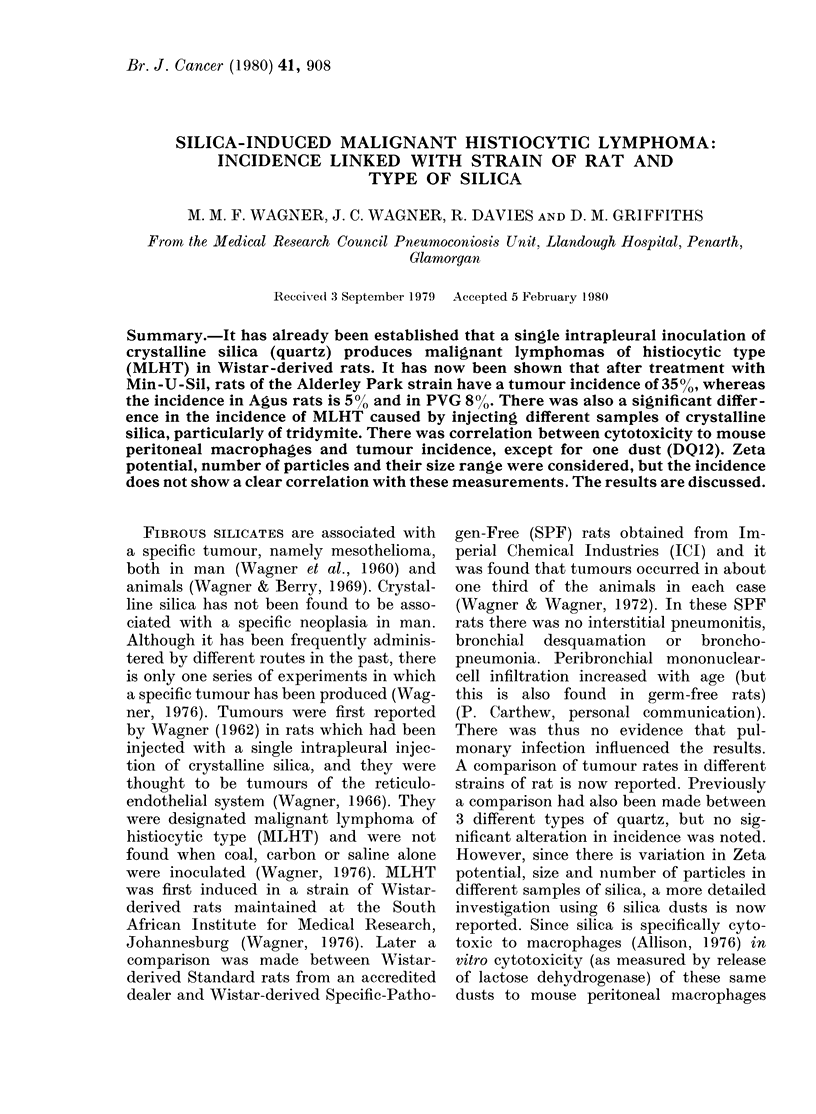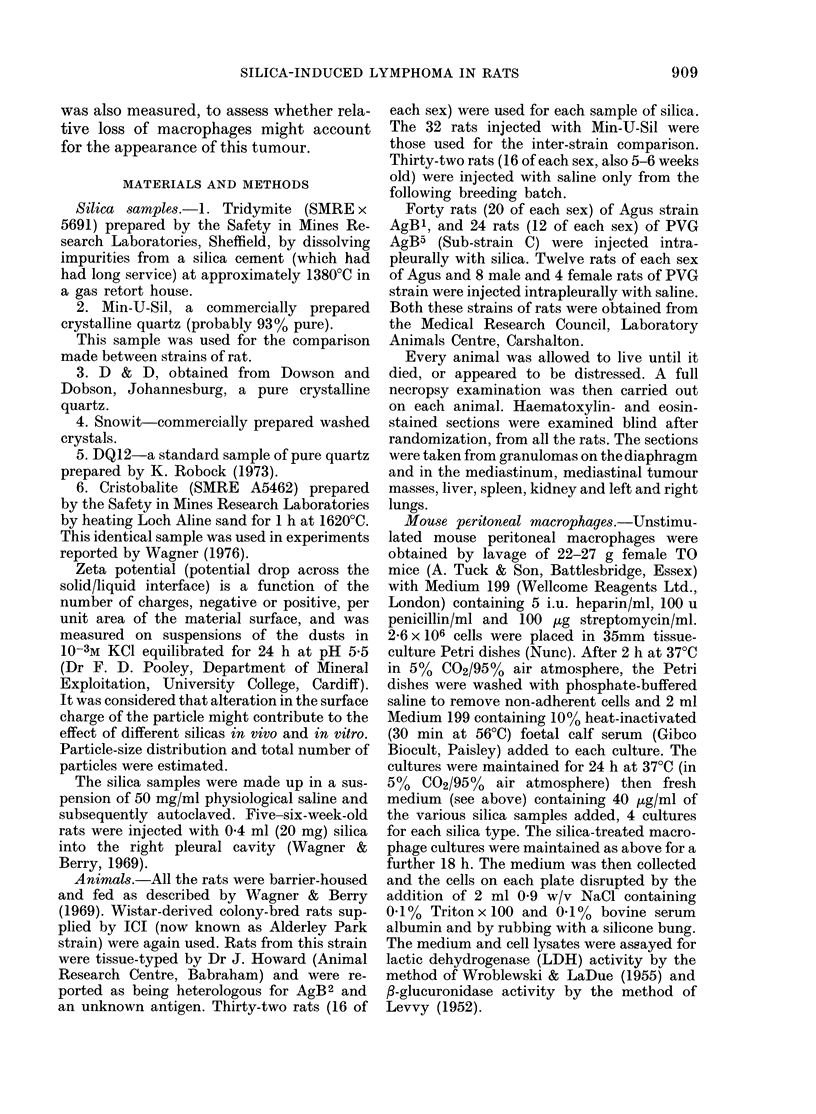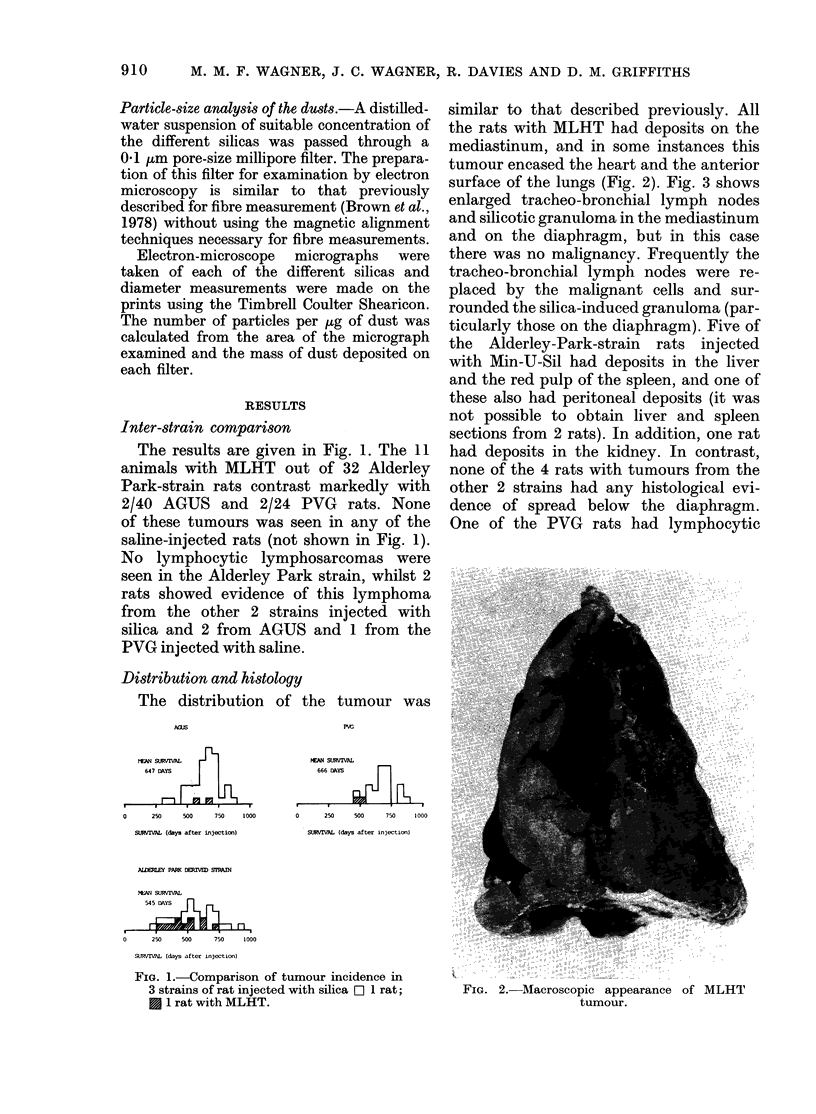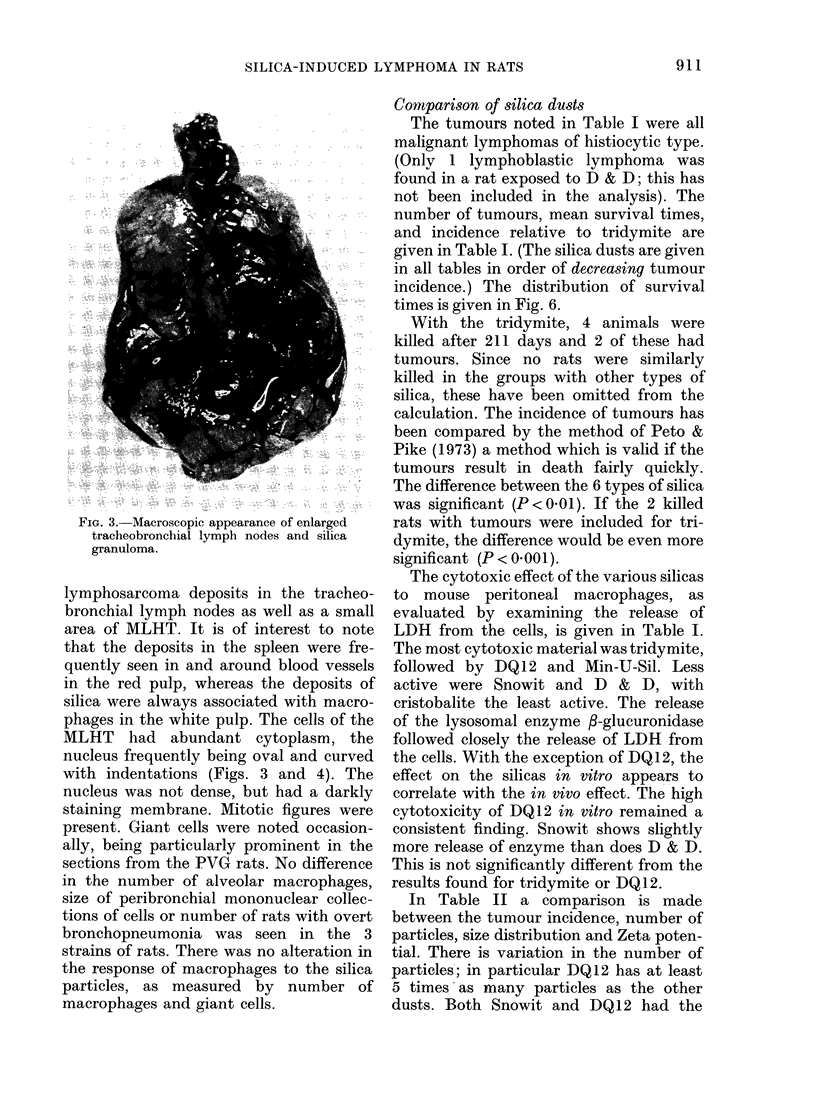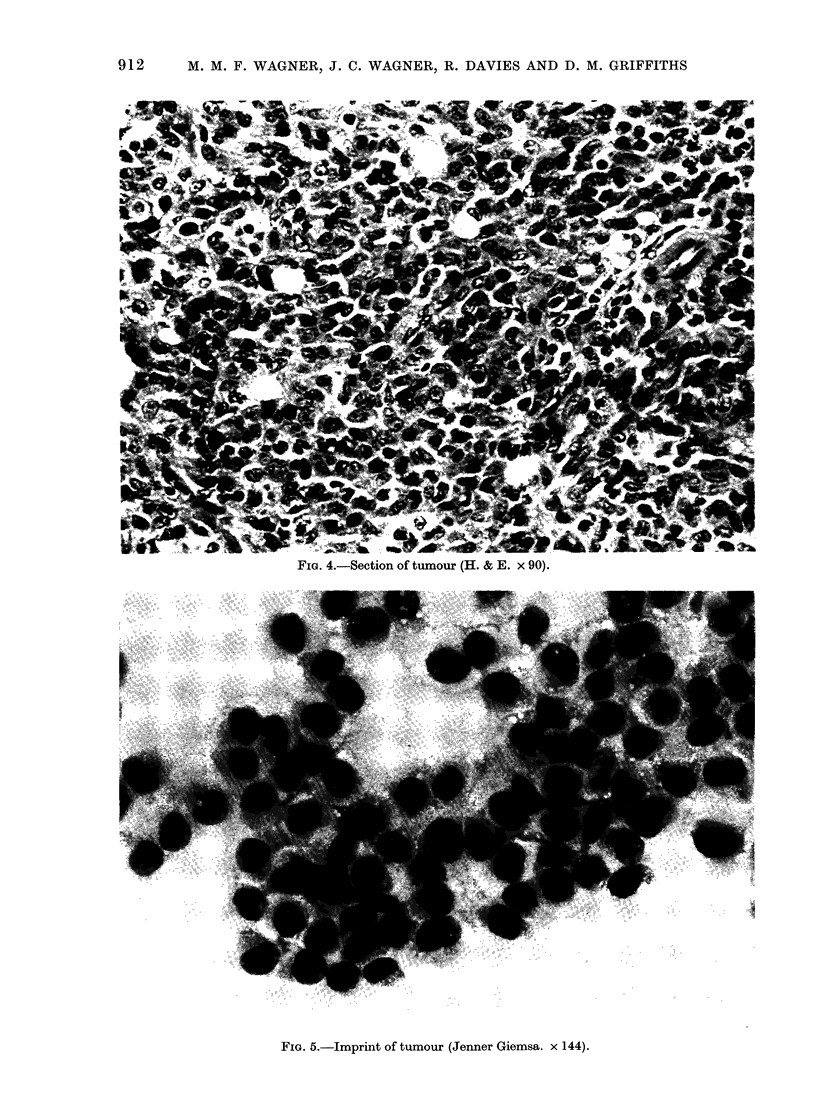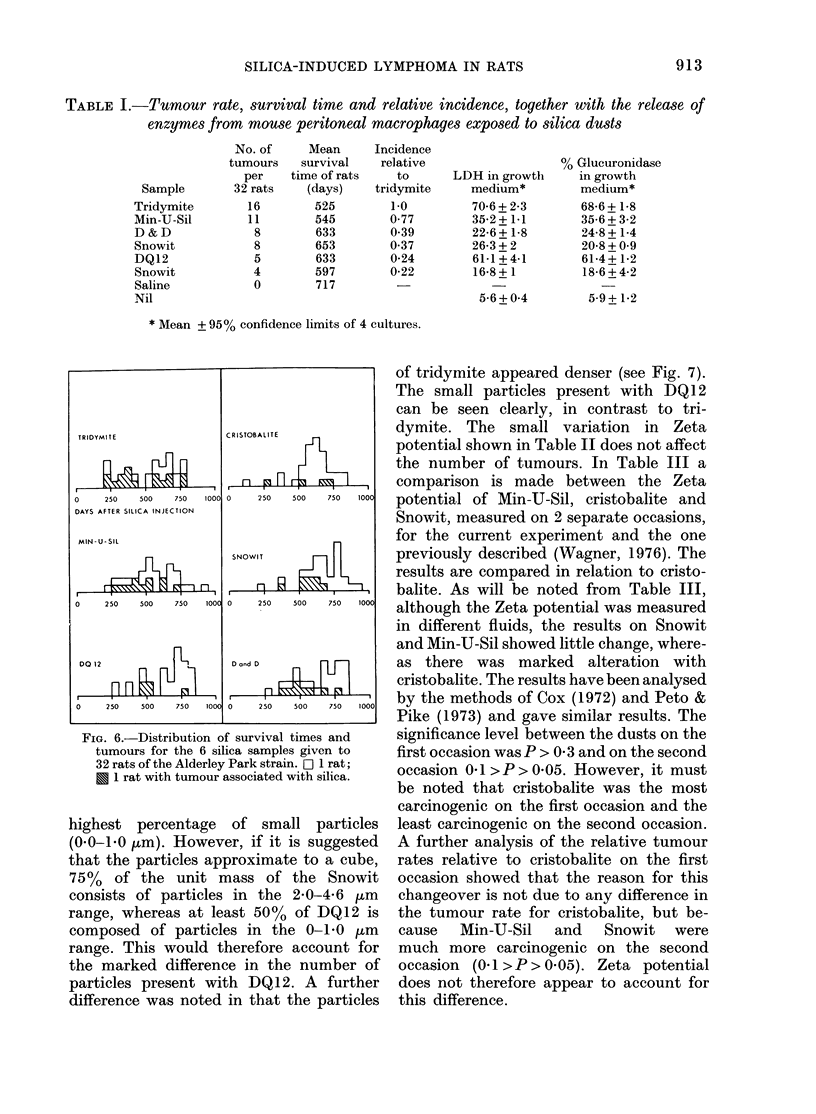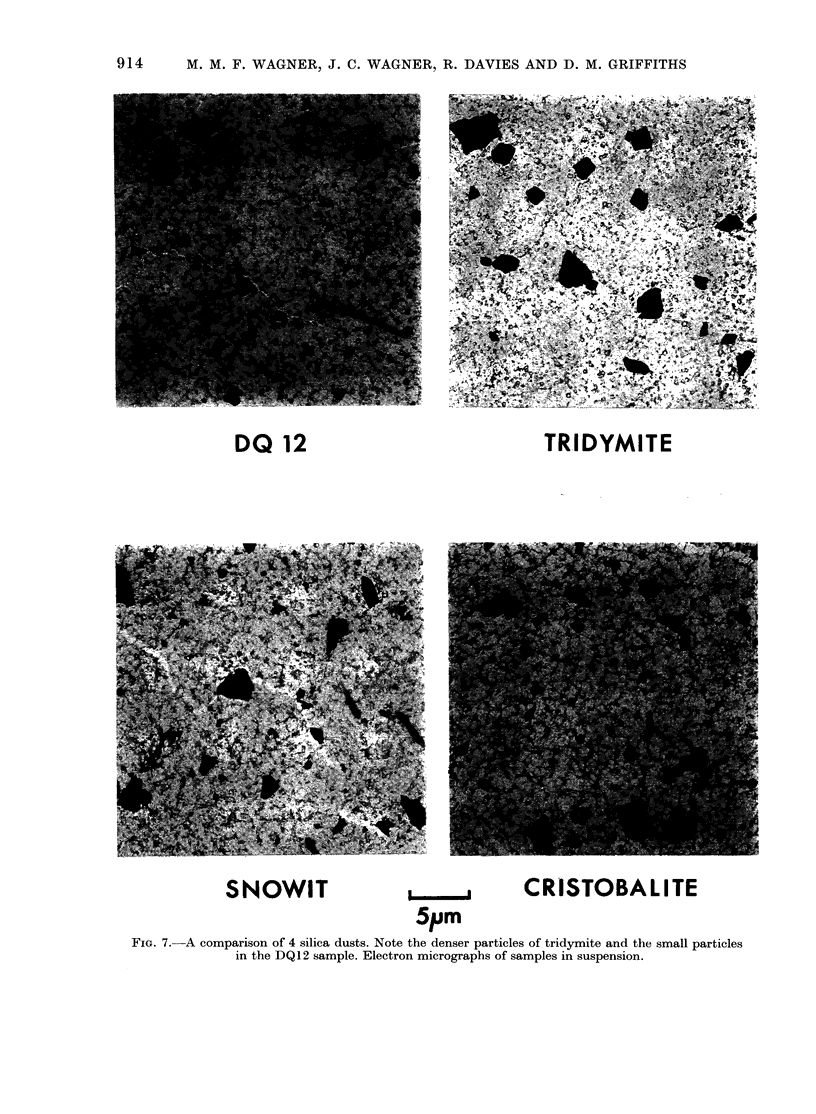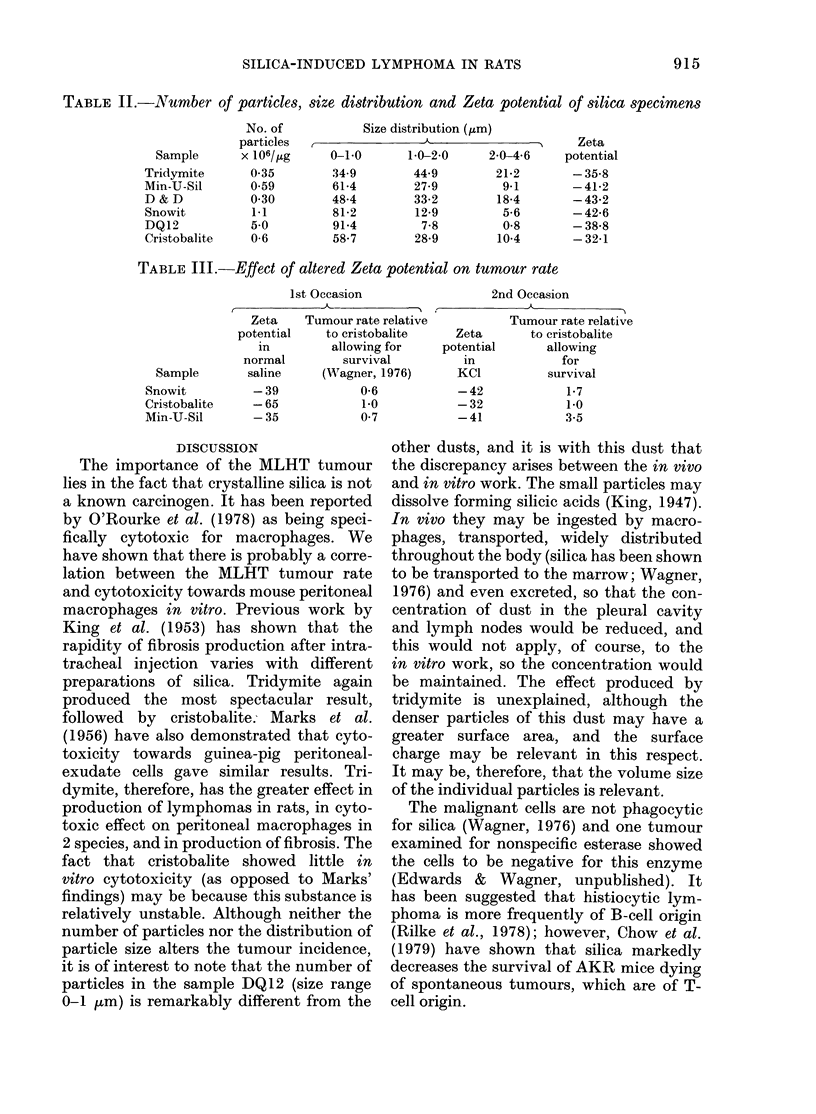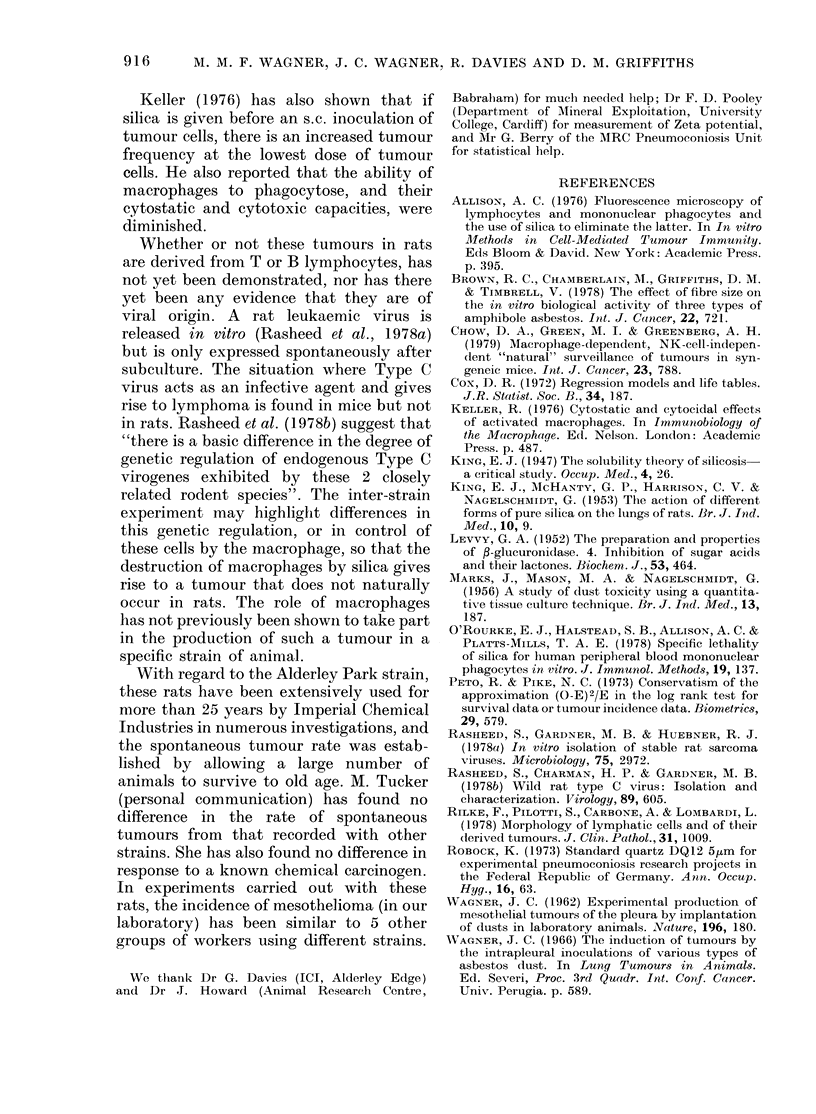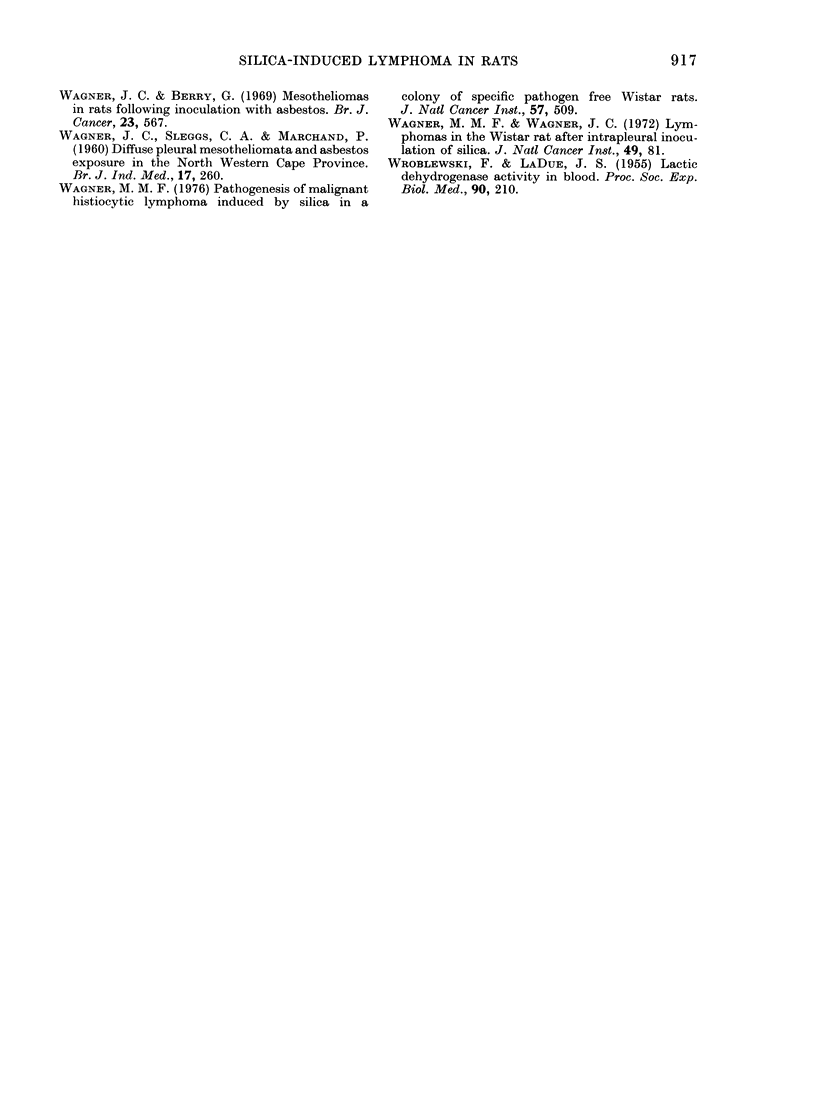# Silica-induced malignant histiocytic lymphoma: incidence linked with strain of rat and type of silica.

**DOI:** 10.1038/bjc.1980.168

**Published:** 1980-06

**Authors:** M. M. Wagner, J. C. Wagner, R. Davies, D. M. Griffiths

## Abstract

**Images:**


*Br. J. Cancer* (1980) **41**, 908

### SILICA-INDUCED MALIGNANT HISTIOCYTIC LYMPHOMA: INCIDENCE LINKED WITH STRAIN OF RAT AND TYPE OF SILICA

M. M. F. WAGNER, J. C. WAGNER, R. DAVIES AND D. M. GRIFFITHS

*From the Medical Research Council Pneumoconiosis Unit, Llandough Hospital, Penarth, Glamorgan*

Received 3 September 1979 Accepted 5 February 1980

**Summary.—It has already been established that a single intrapleural inoculation of crystalline silica (quartz) produces malignant lymphomas of histiocytic type (MLHT) in Wistar-derived rats. It has now been shown that after treatment with Min-U-Sil, rats of the Alderley Park strain have a tumour incidence of 35%, whereas the incidence in Agus rats is 5% and in PVG 8%. There was also a significant difference in the incidence of MLHT caused by injecting different samples of crystalline silica, particularly of tridymite. There was correlation between cytotoxicity to mouse peritoneal macrophages and tumour incidence, except for one dust (DQ12). Zeta potential, number of particles and their size range were considered, but the incidence does not show a clear correlation with these measurements. The results are discussed.**

FIBROUS SILICATES are associated with a specific tumour, namely mesothelioma, both in man (Wagner *et al.*, 1960) and animals (Wagner & Berry, 1969). Crystalline silica has not been found to be associated with a specific neoplasia in man. Although it has been frequently administered by different routes in the past, there is only one series of experiments in which a specific tumour has been produced (Wagner, 1976). Tumours were first reported by Wagner (1962) in rats which had been injected with a single intrapleural injection of crystalline silica, and they were thought to be tumours of the reticuloendothelial system (Wagner, 1966). They were designated malignant lymphoma of histiocytic type (MLHT) and were not found when coal, carbon or saline alone were inoculated (Wagner, 1976). MLHT was first induced in a strain of Wistar-derived rats maintained at the South African Institute for Medical Research, Johannesburg (Wagner, 1976). Later a comparison was made between Wistar-derived Standard rats from an accredited dealer and Wistar-derived Specific-Pathogen-Free (SPF) rats obtained from Imperial Chemical Industries (ICI) and it was found that tumours occurred in about one third of the animals in each case (Wagner & Wagner, 1972). In these SPF rats there was no interstitial pneumonitis, bronchial desquamation or bronchopneumonia. Peribronchial mononuclear-cell infiltration increased with age (but this is also found in germ-free rats) (P. Carthew, personal communication). There was thus no evidence that pulmonary infection influenced the results. A comparison of tumour rates in different strains of rat is now reported. Previously a comparison had also been made between 3 different types of quartz, but no significant alteration in incidence was noted. However, since there is variation in Zeta potential, size and number of particles in different samples of silica, a more detailed investigation using 6 silica dusts is now reported. Since silica is specifically cytotoxic to macrophages (Allison, 1976) *in vitro* cytotoxicity (as measured by release of lactose dehydrogenase) of these same dusts to mouse peritoneal macrophages was also measured, to assess whether relative loss of macrophages might account for the appearance of this tumour.

### MATERIALS AND METHODS

*Silica samples*.—1. Tridymite (SMRE × 5691) prepared by the Safety in Mines Research Laboratories, Sheffield, by dissolving impurities from a silica cement (which had had long service) at approximately 1380°C in a gas retort house.

2. Min-U-Sil, a commercially prepared crystalline quartz (probably 93% pure).

This sample was used for the comparison made between strains of rat.

3. D & D, obtained from Dowson and Dobson, Johannesburg, a pure crystalline quartz.

4. Snowit—commercially prepared washed crystals.

5. DQ12—a standard sample of pure quartz prepared by K. Robock (1973).

6. Cristobalite (SMRE A5462) prepared by the Safety in Mines Research Laboratories by heating Loch Aline sand for 1 h at 1620°C. This identical sample was used in experiments reported by Wagner (1976).

Zeta potential (potential drop across the solid/liquid interface) is a function of the number of charges, negative or positive, per unit area of the material surface, and was measured on suspensions of the dusts in $10^{-3}$M KCl equilibrated for 24 h at pH 5·5 (Dr F. D. Pooley, Department of Mineral Exploitation, University College, Cardiff). It was considered that alteration in the surface charge of the particle might contribute to the effect of different silicas *in vivo* and *in vitro*. Particle-size distribution and total number of particles were estimated.

The silica samples were made up in a suspension of 50 mg/ml physiological saline and subsequently autoclaved. Five–six-week-old rats were injected with 0·4 ml (20 mg) silica into the right pleural cavity (Wagner & Berry, 1969).

*Animals*.—All the rats were barrier-housed and fed as described by Wagner & Berry (1969). Wistar-derived colony-bred rats supplied by ICI (now known as Alderley Park strain) were again used. Rats from this strain were tissue-typed by Dr J. Howard (Animal Research Centre, Babraham) and were reported as being heterologous for $AgB^2$ and an unknown antigen. Thirty-two rats (16 of each sex) were used for each sample of silica. The 32 rats injected with Min-U-Sil were those used for the inter-strain comparison. Thirty-two rats (16 of each sex, also 5–6 weeks old) were injected with saline only from the following breeding batch.

Forty rats (20 of each sex) of Agus strain $AgB^1$, and 24 rats (12 of each sex) of PVG $AgB^5$ (Sub-strain C) were injected intrapleurally with silica. Twelve rats of each sex of Agus and 8 male and 4 female rats of PVG strain were injected intrapleurally with saline. Both these strains of rats were obtained from the Medical Research Council, Laboratory Animals Centre, Carshalton.

Every animal was allowed to live until it died, or appeared to be distressed. A full necropsy examination was then carried out on each animal. Haematoxylin- and eosin-stained sections were examined blind after randomization, from all the rats. The sections were taken from granulomas on the diaphragm and in the mediastinum, mediastinal tumour masses, liver, spleen, kidney and left and right lungs.

*Mouse peritoneal macrophages*.—Unstimulated mouse peritoneal macrophages were obtained by lavage of 22–27 g female TO mice (A. Tuck & Son, Battlesbridge, Essex) with Medium 199 (Wellcome Reagents Ltd., London) containing 5 i.u. heparin/ml, 100 u penicillin/ml and 100 μg streptomycin/ml. $2{\cdot}6 \times 10^6$ cells were placed in 35mm tissue-culture Petri dishes (Nunc). After 2 h at 37°C in 5% $CO_2$/95% air atmosphere, the Petri dishes were washed with phosphate-buffered saline to remove non-adherent cells and 2 ml Medium 199 containing 10% heat-inactivated (30 min at 56°C) foetal calf serum (Gibco Biocult, Paisley) added to each culture. The cultures were maintained for 24 h at 37°C (in 5% $CO_2$/95% air atmosphere) then fresh medium (see above) containing 40 μg/ml of the various silica samples added, 4 cultures for each silica type. The silica-treated macrophage cultures were maintained as above for a further 18 h. The medium was then collected and the cells on each plate disrupted by the addition of 2 ml 0·9 w/v NaCl containing 0·1% Triton × 100 and 0·1% bovine serum albumin and by rubbing with a silicone bung. The medium and cell lysates were assayed for lactic dehydrogenase (LDH) activity by the method of Wroblewski & LaDue (1955) and β-glucuronidase activity by the method of Levvy (1952).

*Particle-size analysis of the dusts.*—A distilled-water suspension of suitable concentration of the different silicas was passed through a 0·1 $\mu$m pore-size millipore filter. The preparation of this filter for examination by electron microscopy is similar to that previously described for fibre measurement (Brown *et al.*, 1978) without using the magnetic alignment techniques necessary for fibre measurements.

Electron-microscope micrographs were taken of each of the different silicas and diameter measurements were made on the prints using the Timbrell Coulter Shearicon. The number of particles per $\mu$g of dust was calculated from the area of the micrograph examined and the mass of dust deposited on each filter.

### RESULTS

### *Inter-strain comparison*

The results are given in Fig. 1. The 11 animals with MLHT out of 32 Alderley Park-strain rats contrast markedly with 2/40 AGUS and 2/24 PVG rats. None of these tumours was seen in any of the saline-injected rats (not shown in Fig. 1). No lymphocytic lymphosarcomas were seen in the Alderley Park strain, whilst 2 rats showed evidence of this lymphoma from the other 2 strains injected with silica and 2 from AGUS and 1 from the PVG injected with saline.

### *Distribution and histology*

The distribution of the tumour was similar to that described previously. All the rats with MLHT had deposits on the mediastinum, and in some instances this tumour encased the heart and the anterior surface of the lungs (Fig. 2). Fig. 3 shows enlarged tracheo-bronchial lymph nodes and silicotic granuloma in the mediastinum and on the diaphragm, but in this case there was no malignancy. Frequently the tracheo-bronchial lymph nodes were replaced by the malignant cells and surrounded the silica-induced granuloma (particularly those on the diaphragm). Five of the Alderley-Park-strain rats injected with Min-U-Sil had deposits in the liver and the red pulp of the spleen, and one of these also had peritoneal deposits (it was not possible to obtain liver and spleen sections from 2 rats). In addition, one rat had deposits in the kidney. In contrast, none of the 4 rats with tumours from the other 2 strains had any histological evidence of spread below the diaphragm. One of the PVG rats had lymphocytic

FIG. 1.—Comparison of tumour incidence in 3 strains of rat injected with silica □ 1 rat; ▨ 1 rat with MLHT.

FIG. 2.—Macroscopic appearance of MLHT tumour.

FIG. 3.—Macroscopic appearance of enlarged tracheobronchial lymph nodes and silica granuloma.

lymphosarcoma deposits in the tracheobronchial lymph nodes as well as a small area of MLHT. It is of interest to note that the deposits in the spleen were frequently seen in and around blood vessels in the red pulp, whereas the deposits of silica were always associated with macrophages in the white pulp. The cells of the MLHT had abundant cytoplasm, the nucleus frequently being oval and curved with indentations (Figs. 3 and 4). The nucleus was not dense, but had a darkly staining membrane. Mitotic figures were present. Giant cells were noted occasionally, being particularly prominent in the sections from the PVG rats. No difference in the number of alveolar macrophages, size of peribronchial mononuclear collections of cells or number of rats with overt bronchopneumonia was seen in the 3 strains of rats. There was no alteration in the response of macrophages to the silica particles, as measured by number of macrophages and giant cells.

*Comparison of silica dusts*

The tumours noted in Table I were all malignant lymphomas of histiocytic type. (Only 1 lymphoblastic lymphoma was found in a rat exposed to D & D; this has not been included in the analysis). The number of tumours, mean survival times, and incidence relative to tridymite are given in Table I. (The silica dusts are given in all tables in order of *decreasing* tumour incidence.) The distribution of survival times is given in Fig. 6.

With the tridymite, 4 animals were killed after 211 days and 2 of these had tumours. Since no rats were similarly killed in the groups with other types of silica, these have been omitted from the calculation. The incidence of tumours has been compared by the method of Peto & Pike (1973) a method which is valid if the tumours result in death fairly quickly. The difference between the 6 types of silica was significant ($P < 0{\cdot}01$). If the 2 killed rats with tumours were included for tridymite, the difference would be even more significant ($P < 0{\cdot}001$).

The cytotoxic effect of the various silicas to mouse peritoneal macrophages, as evaluated by examining the release of LDH from the cells, is given in Table I. The most cytotoxic material was tridymite, followed by DQ12 and Min-U-Sil. Less active were Snowit and D & D, with cristobalite the least active. The release of the lysosomal enzyme $\beta$-glucuronidase followed closely the release of LDH from the cells. With the exception of DQ12, the effect on the silicas *in vitro* appears to correlate with the *in vivo* effect. The high cytotoxicity of DQ12 *in vitro* remained a consistent finding. Snowit shows slightly more release of enzyme than does D & D. This is not significantly different from the results found for tridymite or DQ12.

In Table II a comparison is made between the tumour incidence, number of particles, size distribution and Zeta potential. There is variation in the number of particles; in particular DQ12 has at least 5 times as many particles as the other dusts. Both Snowit and DQ12 had the

FIG. 4.—Section of tumour (H. & E. × 90).

FIG. 5.—Imprint of tumour (Jenner Giemsa. × 144).

TABLE I.—*Tumour rate, survival time and relative incidence, together with the release of enzymes from mouse peritoneal macrophages exposed to silica dusts*

| Sample | No. of tumours per 32 rats | Mean survival time of rats (days) | Incidence relative to tridymite | LDH in growth medium* | % Glucuronidase in growth medium* |
|---|---|---|---|---|---|
| Tridymite | 16 | 525 | 1·0 | 70·6 ± 2·3 | 68·6 ± 1·8 |
| Min-U-Sil | 11 | 545 | 0·77 | 35·2 ± 1·1 | 35·6 ± 3·2 |
| D & D | 8 | 633 | 0·39 | 22·6 ± 1·8 | 24·8 ± 1·4 |
| Snowit | 8 | 653 | 0·37 | 26·3 ± 2 | 20·8 ± 0·9 |
| DQ12 | 5 | 633 | 0·24 | 61·1 ± 4·1 | 61·4 ± 1·2 |
| Snowit | 4 | 597 | 0·22 | 16·8 ± 1 | 18·6 ± 4·2 |
| Saline | 0 | 717 | — | — | — |
| Nil | | | | 5·6 ± 0·4 | 5·9 ± 1·2 |

* Mean ± 95% confidence limits of 4 cultures.

FIG. 6.—Distribution of survival times and tumours for the 6 silica samples given to 32 rats of the Alderley Park strain. □ 1 rat; ▨ 1 rat with tumour associated with silica.

highest percentage of small particles (0·0–1·0 μm). However, if it is suggested that the particles approximate to a cube, 75% of the unit mass of the Snowit consists of particles in the 2·0–4·6 μm range, whereas at least 50% of DQ12 is composed of particles in the 0–1·0 μm range. This would therefore account for the marked difference in the number of particles present with DQ12. A further difference was noted in that the particles of tridymite appeared denser (see Fig. 7). The small particles present with DQ12 can be seen clearly, in contrast to tridymite. The small variation in Zeta potential shown in Table II does not affect the number of tumours. In Table III a comparison is made between the Zeta potential of Min-U-Sil, cristobalite and Snowit, measured on 2 separate occasions, for the current experiment and the one previously described (Wagner, 1976). The results are compared in relation to cristobalite. As will be noted from Table III, although the Zeta potential was measured in different fluids, the results on Snowit and Min-U-Sil showed little change, whereas there was marked alteration with cristobalite. The results have been analysed by the methods of Cox (1972) and Peto & Pike (1973) and gave similar results. The significance level between the dusts on the first occasion was $P > 0{\cdot}3$ and on the second occasion $0{\cdot}1 > P > 0{\cdot}05$. However, it must be noted that cristobalite was the most carcinogenic on the first occasion and the least carcinogenic on the second occasion. A further analysis of the relative tumour rates relative to cristobalite on the first occasion showed that the reason for this changeover is not due to any difference in the tumour rate for cristobalite, but because Min-U-Sil and Snowit were much more carcinogenic on the second occasion ($0{\cdot}1 > P > 0{\cdot}05$). Zeta potential does not therefore appear to account for this difference.

FIG. 7.—A comparison of 4 silica dusts. Note the denser particles of tridymite and the small particles in the DQ12 sample. Electron micrographs of samples in suspension.

TABLE II.—*Number of particles, size distribution and Zeta potential of silica specimens*

| Sample | No. of particles $\times 10^6/\mu g$ | Size distribution ($\mu m$) 0–1·0 | 1·0–2·0 | 2·0–4·6 | Zeta potential |
|---|---|---|---|---|---|
| Tridymite | 0·35 | 34·9 | 44·9 | 21·2 | −35·8 |
| Min-U-Sil | 0·59 | 61·4 | 27·9 | 9·1 | −41·2 |
| D & D | 0·30 | 48·4 | 33·2 | 18·4 | −43·2 |
| Snowit | 1·1 | 81·2 | 12·9 | 5·6 | −42·6 |
| DQ12 | 5·0 | 91·4 | 7·8 | 0·8 | −38·8 |
| Cristobalite | 0·6 | 58·7 | 28·9 | 10·4 | −32·1 |

TABLE III.—*Effect of altered Zeta potential on tumour rate*

| | 1st Occasion | | 2nd Occasion | |
|---|---|---|---|---|
| Sample | Zeta potential in normal saline | Tumour rate relative to cristobalite allowing for survival (Wagner, 1976) | Zeta potential in KCl | Tumour rate relative to cristobalite allowing for survival |
| Snowit | −39 | 0·6 | −42 | 1·7 |
| Cristobalite | −65 | 1·0 | −32 | 1·0 |
| Min-U-Sil | −35 | 0·7 | −41 | 3·5 |

DISCUSSION

The importance of the MLHT tumour lies in the fact that crystalline silica is not a known carcinogen. It has been reported by O'Rourke *et al.* (1978) as being specifically cytotoxic for macrophages. We have shown that there is probably a correlation between the MLHT tumour rate and cytotoxicity towards mouse peritoneal macrophages *in vitro*. Previous work by King *et al.* (1953) has shown that the rapidity of fibrosis production after intratracheal injection varies with different preparations of silica. Tridymite again produced the most spectacular result, followed by cristobalite. Marks *et al.* (1956) have also demonstrated that cytotoxicity towards guinea-pig peritoneal-exudate cells gave similar results. Tridymite, therefore, has the greater effect in production of lymphomas in rats, in cytotoxic effect on peritoneal macrophages in 2 species, and in production of fibrosis. The fact that cristobalite showed little *in vitro* cytotoxicity (as opposed to Marks' findings) may be because this substance is relatively unstable. Although neither the number of particles nor the distribution of particle size alters the tumour incidence, it is of interest to note that the number of particles in the sample DQ12 (size range 0–1 $\mu m$) is remarkably different from the other dusts, and it is with this dust that the discrepancy arises between the *in vivo* and *in vitro* work. The small particles may dissolve forming silicic acids (King, 1947). *In vivo* they may be ingested by macrophages, transported, widely distributed throughout the body (silica has been shown to be transported to the marrow; Wagner, 1976) and even excreted, so that the concentration of dust in the pleural cavity and lymph nodes would be reduced, and this would not apply, of course, to the *in vitro* work, so the concentration would be maintained. The effect produced by tridymite is unexplained, although the denser particles of this dust may have a greater surface area, and the surface charge may be relevant in this respect. It may be, therefore, that the volume size of the individual particles is relevant.

The malignant cells are not phagocytic for silica (Wagner, 1976) and one tumour examined for nonspecific esterase showed the cells to be negative for this enzyme (Edwards & Wagner, unpublished). It has been suggested that histiocytic lymphoma is more frequently of B-cell origin (Rilke *et al.*, 1978); however, Chow *et al.* (1979) have shown that silica markedly decreases the survival of AKR mice dying of spontaneous tumours, which are of T-cell origin.

Keller (1976) has also shown that if silica is given before an s.c. inoculation of tumour cells, there is an increased tumour frequency at the lowest dose of tumour cells. He also reported that the ability of macrophages to phagocytose, and their cytostatic and cytotoxic capacities, were diminished.

Whether or not these tumours in rats are derived from T or B lymphocytes, has not yet been demonstrated, nor has there yet been any evidence that they are of viral origin. A rat leukaemic virus is released *in vitro* (Rasheed *et al.*, 1978*a*) but is only expressed spontaneously after subculture. The situation where Type C virus acts as an infective agent and gives rise to lymphoma is found in mice but not in rats. Rasheed *et al.* (1978*b*) suggest that "there is a basic difference in the degree of genetic regulation of endogenous Type C virogenes exhibited by these 2 closely related rodent species". The inter-strain experiment may highlight differences in this genetic regulation, or in control of these cells by the macrophage, so that the destruction of macrophages by silica gives rise to a tumour that does not naturally occur in rats. The role of macrophages has not previously been shown to take part in the production of such a tumour in a specific strain of animal.

With regard to the Alderley Park strain, these rats have been extensively used for more than 25 years by Imperial Chemical Industries in numerous investigations, and the spontaneous tumour rate was established by allowing a large number of animals to survive to old age. M. Tucker (personal communication) has found no difference in the rate of spontaneous tumours from that recorded with other strains. She has also found no difference in response to a known chemical carcinogen. In experiments carried out with these rats, the incidence of mesothelioma (in our laboratory) has been similar to 5 other groups of workers using different strains.

We thank Dr G. Davies (ICI, Alderley Edge) and Dr J. Howard (Animal Research Centre, Babraham) for much needed help; Dr F. D. Pooley (Department of Mineral Exploitation, University College, Cardiff) for measurement of Zeta potential, and Mr G. Berry of the MRC Pneumoconiosis Unit for statistical help.

### REFERENCES

ALLISON, A. C. (1976) Fluorescence microscopy of lymphocytes and mononuclear phagocytes and the use of silica to eliminate the latter. In *In vitro Methods in Cell-Mediated Tumour Immunity.* Eds Bloom & David. New York: Academic Press. p. 395.

BROWN, R. C., CHAMBERLAIN, M., GRIFFITHS, D. M. & TIMBRELL, V. (1978) The effect of fibre size on the *in vitro* biological activity of three types of amphibole asbestos. *Int. J. Cancer*, **22**, 721.

CHOW, D. A., GREEN, M. I. & GREENBERG, A. H. (1979) Macrophage-dependent, NK-cell-independent "natural" surveillance of tumours in syngeneic mice. *Int. J. Cancer*, **23**, 788.

COX, D. R. (1972) Regression models and life tables. *J.R. Statist. Soc. B.*, **34**, 187.

KELLER, R. (1976) Cytostatic and cytocidal effects of activated macrophages. In *Immunobiology of the Macrophage.* Ed. Nelson. London: Academic Press. p. 487.

KING, E. J. (1947) The solubility theory of silicosis—a critical study. *Occup. Med.*, **4**, 26.

KING, E. J., MCHANTY, G. P., HARRISON, C. V. & NAGELSCHMIDT, G. (1953) The action of different forms of pure silica on the lungs of rats. *Br. J. Ind. Med.*, **10**, 9.

LEVVY, G. A. (1952) The preparation and properties of β-glucuronidase. 4. Inhibition of sugar acids and their lactones. *Biochem. J.*, **53**, 464.

MARKS, J., MASON, M. A. & NAGELSCHMIDT, G. (1956) A study of dust toxicity using a quantitative tissue culture technique. *Br. J. Ind. Med.*, **13**, 187.

O'ROURKE, E. J., HALSTEAD, S. B., ALLISON, A. C. & PLATTS-MILLS, T. A. E. (1978) Specific lethality of silica for human peripheral blood mononuclear phagocytes *in vitro*. *J. Immunol. Methods*, **19**, 137.

PETO, R. & PIKE, N. C. (1973) Conservatism of the approximation $(O\text{-}E)^2/E$ in the log rank test for survival data or tumour incidence data. *Biometrics*, **29**, 579.

RASHEED, S., GARDNER, M. B. & HUEBNER, R. J. (1978*a*) *In vitro* isolation of stable rat sarcoma viruses. *Microbiology*, **75**, 2972.

RASHEED, S., CHARMAN, H. P. & GARDNER, M. B. (1978*b*) Wild rat type C virus: Isolation and characterization. *Virology*, **89**, 605.

RILKE, F., PILOTTI, S., CARBONE, A. & LOMBARDI, L. (1978) Morphology of lymphatic cells and of their derived tumours. *J. Clin. Pathol.*, **31**, 1009.

ROBOCK, K. (1973) Standard quartz DQ12 5μm for experimental pneumoconiosis research projects in the Federal Republic of Germany. *Ann. Occup. Hyg.*, **16**, 63.

WAGNER, J. C. (1962) Experimental production of mesothelial tumours of the pleura by implantation of dusts in laboratory animals. *Nature*, **196**, 180.

WAGNER, J. C. (1966) The induction of tumours by the intrapleural inoculations of various types of asbestos dust. In *Lung Tumours in Animals.* Ed. Severi, *Proc. 3rd Quadr. Int. Conf. Cancer.* Univ. Perugia. p. 589.

WAGNER, J. C. & BERRY, G. (1969) Mesotheliomas in rats following inoculation with asbestos. *Br. J. Cancer*, **23**, 567.

WAGNER, J. C., SLEGGS, C. A. & MARCHAND, P. (1960) Diffuse pleural mesotheliomata and asbestos exposure in the North Western Cape Province. *Br. J. Ind. Med.*, **17**, 260.

WAGNER, M. M. F. (1976) Pathogenesis of malignant histiocytic lymphoma induced by silica in a colony of specific pathogen free Wistar rats. *J. Natl Cancer Inst.*, **57**, 509.

WAGNER, M. M. F. & WAGNER, J. C. (1972) Lymphomas in the Wistar rat after intrapleural inoculation of silica. *J. Natl Cancer Inst.*, **49**, 81.

WROBLEWSKI, F. & LADUE, J. S. (1955) Lactic dehydrogenase activity in blood. *Proc. Soc. Exp. Biol. Med.*, **90**, 210.